# Operative Surgery, Based on Normal and Pathological Anatomy

**Published:** 1852-06

**Authors:** 


*Operative Surgery, based on Normal and Pathological Anatomy.* By J. F. MALGAIGNE, Professor Agrege de la Faculte de Medecine de Paris, Chirurgien de l'Hopital de Dourcine, Chevalier de la Legion d'Honneur, et du Merite Militaire de Pologne, etc. etc. Translated from the French by FREDERICK BRITTAN, M. D. Illustrated by Wood Engravings, from Designs by WESTMACOTT. Philadelphia: Blanchard & Lea. 1851. For sale by Derby, 164 Main-st., Buffalo.

The original work "*Manuel de Medicine Operatoire*," was published in 1843, and we have long been accustomed to refer to it as one of the most valuable text books in our library. It has been translated into at least five continental languages, and in Paris, where it has already reached a fourth edition, it is a very general text book among the medical students of all nations.

We have, therefore, often expressed our surprise that it was not, before this, translated into the English.

To those who have ever seen "Malgaigne's Operative Surgery," it is superfluous to say that it is a valuable book. Although somewhat less elaborate, it occupies a place with Velpeau, Chelius, Lisfranc, &c.

The writings of Lisfranc, Nelaton and others, still wait for a translator. We wish they might fall into the hands of Dr. Brittain. F. H. H.

